# Positive changes to written language following phonological treatment in logopenic variant primary progressive aphasia: Case report

**DOI:** 10.3389/fnhum.2022.1006350

**Published:** 2023-01-25

**Authors:** Katlyn Nickels, Pélagie M. Beeson, Kindle Rising, Fatima Jebahi, Aneta Kielar

**Affiliations:** ^1^Department of Speech, Language, and Hearing Sciences, The University of Arizona, Tucson, AZ, United States; ^2^Department of Neurology, The University of Arizona, Tucson, AZ, United States

**Keywords:** case report, logopenic variant primary progressive aphasia, phonological treatment, written language, tDCS

## Abstract

Phonological impairment contributes to deficits in repetition and spoken naming in logopenic variant Primary Progressive Aphasia (lvPPA), but weakened phonology can also affect written language skills. In this experimental case report, we demonstrate phonological text agraphia in a 71-year-old woman in the early stages of lvPPA that undermined her ability to write meaningful, grammatical sentences. We investigated the therapeutic value of a rigorous treatment protocol to strengthen phonological manipulation skills coupled with transcranial direct current stimulation (tDCS). Intervention took place 5 days a week for 2 weeks with active tDCS, followed by a 2-month rest period, and then a second period of phonological treatment with sham tDCS. Over the course of treatment, our participant demonstrated improved phonological transcoding and manipulation skills as well as marked improvement in the proportion of grammatically well-formed, meaningful written narratives. Improvements in spelling and letter selection were also observed. Treatment gains were documented during phonological intervention in both active tDCS and sham treatment phases and were maintained 2 months after the conclusion of intervention. Importantly, improvements were observed in the context of a progressive disorder. These data present compelling evidence regarding the impairment-based approach that targets compromised phonological skills, presenting opportunity for improving functional written communication skills relevant to the everyday lives of individuals with lvPPA.

## Introduction

The logopenic variant of primary progressive aphasia (lvPPA), one of three recognized PPA subtypes, is associated with atrophy in left posterior perisylvian regions (Gorno-Tempini et al., 2004, 2008; Grossman, 2010). The core characteristics of this PPA variant include impaired word retrieval and impaired repetition of phrases or sentences with relatively spared speech production and grammatical construction (Gorno-Tempini et al., 2011). These spoken language features reflect disruption of the posterior dorsal articulatory/phonological pathway in a manner similar to stroke-related aphasia following temporoparietal damage (Keator et al., 2021). Whereas articulatory skill is typically preserved and spoken output is considered fluent, errors in phonological assembly may result in phonemic paraphasias. Furthermore, when underlying cognitive/linguistic abilities are evaluated using tasks that require phonological manipulation skills, they are clearly impaired in lvPPA. For these reasons, the logopenic variant has also been referred to as phonological PPA (Gorno-Tempini et al., 2008; Henry et al., 2016; Cotelli et al., 2020).

The functional consequence of the underlying phonological impairment in lvPPA has been most obvious in word retrieval difficulties that are ubiquitous in most individuals with PPA, however, written language abilities are also impaired (Sepelyak et al., 2011; Shim et al., 2012; Faria et al., 2013; Graham, 2014). Henry et al. (2012, 2016) demonstrated classic phonological alexia/agraphia patterns in lvPPA characterized by disproportionately poor performance on non-words in relation to real words. Real word spelling was also impaired, and spelling was worse than reading, overall. This profile is similar to that of individuals with stroke-related phonological impairment (Henry et al., 2007; Rapcsak et al., 2009; Beeson et al., 2022). The effects of the underlying phonological impairment on text-level reading and writing has received limited attention in lvPPA, but a number of case reports have made note of impaired written communication (Trebbastoni et al., 2013; Tsapkini and Hillis, 2013; Ficek et al., 2018). In those with stroke-related phonological impairment, Friedman (1995) documented marked difficulty reading grammatical words (functors) and morphological markers, a profile termed phonological text alexia. By analogy, Beeson et al. (2018) characterized the complementary phonological text agraphia profile in which individuals with relatively well-recovered spoken language but persistent impairment of phonological skill had marked difficulty generating written sentences that were well-formed and grammatically correct. It follows that phonological text agraphia may be a relatively early sign of phonological impairment in those with lvPPA, but this has not been documented.

Based on a recent meta-analysis of oral and written naming (Cotelli et al., 2020), about 50 studies have examined behavioral treatments for PPA. In this systematic review, Cotelli et al. (2020) cited 47 studies, including 16 that examined individuals with lvPPA. As with all PPA subtypes, most treatments for the logopenic variant have focused on improving lexical retrieval skills with spoken naming as the primary outcome variable, and some have included written naming. The treatment protocols have ranged from repeated practice of spoken words paired with written words (e.g., Meyer et al., 2017; Croot et al., 2019) to more cognitively demanding tasks that train self-cuing strategies (e.g., Henry et al., 2013; Beales et al., 2016; Kim, 2017; Dial et al., 2019; Grasso et al., 2019), and treatments that also engage executive functions to guide generative naming (e.g., Beeson et al., 2011; de Aguiar et al., 2022). Overall, lexical retrieval treatments have consistently shown positive outcomes for trained items, but they vary regarding generalization and maintenance effects (Cotelli et al., 2020). Similarly, treatments directed toward relearning spelling of targeted items have demonstrated good outcomes (e.g., Ficek et al., 2018; Tsapkini et al., 2018). Of particular relevance to lvPPA is treatment directed toward strengthening weakened sound-letter correspondences that also resulted in positive outcomes (Tsapkini and Hillis, 2013; Tsapkini et al., 2014). Thus, treatment outcomes for lvPPA have been promising, affirming that residual components of the left lateralized language network can be leveraged to improve language function. To our knowledge, there have been no treatment studies with PPA targeting the underlying phonological manipulation skills that are vulnerable with damage to the dorsal language network, which was the focus of this study.

Transcranial direct current stimulation (tDCS) has emerged as a strong candidate approach in the effort to maximize the effects of behavioral rehabilitation in PPA (for recent reviews see Nissim et al., 2020; Coemans et al., 2021). In relation to other neuromodulatory approaches, tDCS advantages include good tolerance and portability for clinical use (Nissim et al., 2020). The non-invasive application of electrical current *via* scalp electrodes is thought to increase neuronal excitability in the vicinity of anodal placement and decrease excitability near the cathode. Placement of anodal and cathodal electrodes is important because modulation of excitatory and inhibitory influences across the right and left hemisphere regions may affect linguistic and non-linguistic performance. Thus, the cognitive task(s), electrode locations, and other dosage considerations are relevant for predicting and interpreting treatment effects.

Across the range of PPA subtypes, anodal electrode placement has been examined in temporoparietal regions (Hung et al., 2017; Roncero et al., 2017; Shah-Basak et al., 2022), dorsolateral prefrontal cortex (Cotelli et al., 2014, 2016), and the inferior frontal lobe (Ficek et al., 2018; Tsapkini et al., 2018; de Aguiar et al., 2022). In the context of lvPPA, treatment may logically be directed toward strengthening weakened phonological processes associated with temporoparietal atrophy. With this in mind, anodal stimulation to frontal components of the dorsal articulatory/phonological network is well justified as neuroimaging studies have demonstrated activation in this region during reading and spelling (Beeson et al., 2003; Purcell et al., 2011; Planton et al., 2013) and sublexical phonological tasks (Burton et al., 2000; DeMarco et al., 2018). Consistent with this logic, Tsapkini et al. (2014) demonstrated positive effects of anodal stimulation to left inferior frontal lobe paired with retraining of sound-letter correspondences in a mixed group of individuals with PPA. In addition, Tsapkini et al. (2018) documented benefits of tDCS paired with a lexical spelling treatment finding that improvements in trained and untrained items were best maintained after active tDCS.

In the present study, we examined the effects of intensive phonological treatment for an individual with lvPPA paired with tDCS targeting the left inferior frontal gyrus. We hypothesized that this approach would enhance function of relevant task-related brain networks and lead to improvements in phonological processing with generalization to written language. Here we report treatment outcomes from an individual case to demonstrate the therapeutic value of the approach during treatment phases with active and sham tDCS.

### Case report

Our participant (LV2) was a 71-year-old, right-handed, native English speaker with 16 years of education. Prior to retirement, she worked as an elementary school teacher. She was referred to us 3 months after receiving a diagnosis of lvPPA from a neurologist based on results from neuroimaging and neuropsychological evaluation. A PET scan revealed asymmetric temporoparietal hypometabolism more pronounced in the left hemisphere, consistent with lvPPA. During our initial assessment, LV2 reported that she had been experiencing language difficulty for approximately 15 months. Her primary complaints were trouble with word-finding, spelling, and “remembering instructions” (e.g., following a recipe). LV2’s health history was otherwise unremarkable, and she had not previously received any speech-language services. LV2’s language impairment was confirmed by performance on the Western Aphasia Battery-Revised (WAB-R; Kertesz, 2007), revealing a profile consistent with anomic aphasia and an Aphasia Quotient (AQ) of 87. Composite scores reflected mild deficits of repetition (8.8/10) and auditory comprehension (7.7/10). The naming deficit was evident on WAB-R subtests (7.6/10) and was also pronounced on the Boston Naming Test (BNT; Kaplan et al., 1983) with 23 of 60 correct. LV2’s conversational speech and narrative description of the WAB-R picnic scene also revealed marked word-finding difficulties with unimpaired speech production and relatively preserved grammatical construction of sentences. When asked to produce a written description of the picnic scene from the WAB-R in a manner comparable to the spoken task, she produced agrammatic attempts at sentences with many spelling errors. The striking contrast by modality is illustrated by LV2’s spoken comment about the picture, “This is a tree with a lot of beautiful growth on it” in comparison to her written attempt at a similar thought, *Tree wt fowers*.

To formally characterize LV2’s cognitive and speech/language abilities, as well as document her pattern of cortical atrophy, seventeen right-handed neurotypical adults (5 males, 12 females) were recruited for this study to provide behavioral and neuroimaging comparison data. The control cohort was comparable to LV2 regarding age [*t*(16) = 0.04, *p* = 0.972] and education [*t*(16) = –0.41, *p* = 0.690]. All were native speakers of English with normal or corrected-to-normal hearing and vision. They had no history of neurological, psychiatric, speech, language, or learning disorders, and were not taking neuroleptic or mood-altering medications. This cohort performed within normal limits on the Montreal Cognitive Assessment (Nasreddine et al., 2005) with an average score of 26.6 of 30 (SD = 2.12) as well as other standardized neuropsychological tests used in this study (Table 1). Comparisons between LV2 and controls were tested using the modified one-tailed *t*-test designed for single-case studies with procedures described by Crawford and Howell (1998) using the on-line statistical toolbox singlims.exe (Crawford and Garthwaite, 2002).

**TABLE 1:** LV2’s pre-treatment performance with comparison to healthy controls (*n* = 17).

		*M*	SD	*t*	Z-CC	(95% CI)
**Lexical retrieval**						
Boston naming test (%)	38	95.39	4.35	–12.82***	–13.19	(–17.73 to –8.64)
Category fluency (scaled)^1^	1	14.35	3.10	–4.19***	–4.31	(–5.85 to –2.75)
Letter fluency (scaled)^1^	10	14.53	1.66	–2.65*	–2.73	(–3.77 to –1.67)
**Reading and spelling (%)^2^**						
Read words	95	99.71	0.83	–5.51***	–5.67	(–7.67 to –3.66)
Read non-words	90	99.38	1.71	–5.33***	–5.49	(–7.49 to –3.48)
Spell words	80	99.41	1.66	–11.36***	–11.69	(–15.71 to –7.65)
Spell non-words	60	90.62	7.27	–4.09***	–4.21	(–5.78 to –2.64)
**Visual-orthographic (%)**						
Letter reversal P18^3^	100	98.69	1.99	0.64	0.66	(0.12 to 1.18)
Case matching P19^3^	100	99.77	0.93	0.24	0.25	(–0.24 to 0.73)
Visual lexical decision P25^3^	98	98.82	1.84	–0.43	–0.45	(–0.94 to 0.06)
**Auditory processing (%)**						
Minimal pair judgement P1, P2^3^	98	99.53	2.66	–0.56	–0.58	(–1.08 to –0.05)
Rhyme judgement P15^3^	100	98.09	2.73	0.32	0.33	(–0.16 to 0.82)
**Speech production (%)**						
Word repetition^2^	100	100	0			
Non-word repetition^4^	88	91.18	7.61	–0.41	–0.42	(–0.91 to 0.09)
**Phonological processing (%)**						
Rhyme production^5^	100	99.33	2.58	0.25	0.26	(–0.228 to 0.739)
Manipulation^5^						
Sound segmentation	90	98.83	1.80	–4.78***	–4.91	(–6.66 to –3.16)
Sound blending	65	85.88	12.02	–1.69	–1.74	(–2.49 to –0.96)
Sound replacement	40	87.84	11.24	–4.14***	–4.26	(–5.79 to –2.71)
Transcoding^5^						
Letter-to-sound	95	99.71	1.21	–3.78**	–3.89	(–5.30 to –2.47)
Sound-to-letter	65	97.94	5.32	–6.02***	–6.19	(–8.36 to –4.01)
Read CVC non-words	90	98.13	3.10	–2.55**	–2.63	(–3.64 to –1.60)
Spell CVC non-words	65	94.69	6.45	–4.47***	–4.60	(–6.25 to –2.95)
**Semantic processing (%)**						
Peabody picture vocab test	91	96.49	1.92	–2.78*	–2.86	(–3.94 to –1.76)
Camel and Cactus Test	61	91.27	4.59	–6.41***	–6.60	(–8.90 to –4.28)
Written word-picture P48^3^	85	99.56	0.98	–14.49***	–14.86	(–19.97 to –9.74)
Synonym judgement P49^3^	87	97.50	2.19	–4.66***	–4.80	(–6.50 to –3.08)
**Memory, executive function, and visuospatial skills**						
Warrington memory (scaled)	5	7.63	2.16	–1.18	–1.22	(–1.86 to –0.55)
Trail-making^1^ (Scaled)	2	11.93	2.63	–3.66**	–3.78	(–5.24 to –2.30)
Complex fig recall^6^ (scaled)	4	11.88	2.18	–3.51**	–3.61	(–4.94 to –2.28)
Digit span forward (scaled)^7^	10	11.18	2.83	–0.41	–0.42	(–0.91 to 0.09)
Digit span backward (scaled)^7^	7	10.88	3.50	–1.08	–1.11	(–1.71 to –0.49)

^1^Delis–Kaplan executive function system.

^2^Arizona battery for reading and spelling.

^3^Psycholinguistic assessments of language processing in aphasia.

^4^Children’s non-word repetition test.

^5^Arizona phonological battery.

^6^Kaplan-Baycrest neurocognitive assessment.

^7^Wechsler Adult Intelligence Scale—4th ed.

Z-CC = Effect Size for difference between case and controls.

Bold values represent the *t = 0.05, **t = 0.01, ***t = 0.001.

### Behavioral profile before treatment

LV2 completed a comprehensive assessment of speech/language abilities and overall cognitive performance (Table 1). In addition to the confrontation naming impairment document above, LV2 was also impaired on verbal fluency tasks. Repetition of real words was unimpaired whereas non-word repetition was 88%, reflecting a significant lexicality effect (χ^2^ = 10.73, *p* < 0.001). Administration of a motor speech evaluation from Duffy (2006) revealed no dysarthria or apraxia of speech.

Measures of visual and auditory processing affirmed LV2 did not have peripheral impairments that would affect performance (Table 1). Her single word reading was relatively spared (95% correct), and she read the standard Rainbow Passage (Fairbanks, 1960) aloud without errors. Single-word spelling was impaired to some extent (80% correct) with some errors in letter selection suggesting a mild allographic impairment. LV2 showed a mild deficit in non-word reading (90% correct) and a greater impairment of non-word spelling (60% correct). There was a lexicality effect for spelling, with real words (80%) better than non-words (60%), χ^2^ = 8.6, *p* = 0.003.

Regarding phonological processing, LV2’s spoken rhyme production was unimpaired. In contrast, phonological manipulation tasks from the Arizona Phonological Battery (Beeson et al., 2010) were challenging, showing significant impairment on sound segmentation and sound replacement. LV2 also showed mild impairment in transcoding from letters to sounds (90% correct) and reading aloud consonant-vowel-consonant (CVC) non-words (90% correct). Sound-to-letter transcoding was more difficult for LV2, with 65% accuracy for individual sounds and writing CVC non-words.

Measures of conceptual knowledge revealed some impairment of semantics as shown in Table 1 for the Peabody Picture Vocabulary Test (Dunn and Dunn, 2007), Camels and Cactus Test (Adlam et al., 2010), and subtests 48 (written word-to-picture match) and 49 (synonym judgment) from the Psycholinguistic Assessment of Language Processing (Kay et al., 1996).

Regarding tests of non-verbal cognitive skills, LV2 performed within normal limits on the Warrington Memory Test for Faces (Warrington, 1984), but showed impairment on the Trail-Making Test from the Delis-Kaplan Executive Function System (Delis et al., 2001) and the complex figure recall test from the Kaplan-Baycrest Neurocognitive Assessment (Leach, 2010). Performance on digit span was comparable to controls (Wechsler, 2009).

As noted above, LV2’s marked difficulty writing at the sentence/paragraph level was one of her most obvious functional impairments. To better characterize performance, we analyzed her spoken and written picture descriptions in a consistent manner using procedures adapted from spoken narrative analysis by Nicholas and Brookshire (1993). This included calculation of total words (excluding unintelligible/illegible responses), correct information units (CIUs), and informativeness (CIUs/total words). A CIU was defined as a word that was recognizable in context and accurate regarding the content. Informativeness was derived by dividing the number of CIUs by the number of words. For written narratives, we also determined the proportion of CIUs that were spelled correctly, and finally we derived an index of written accuracy as follows: #CIUs–(#spelling errors + #morphological errors)/(#words + #paragraphias). Each written string of words was judged as to whether it constituted a well-formed and complete sentence (i.e., contained appropriate subject, verb, object, and functors), using the same guidelines as Beeson et al. (2018). Transcription and CIU analysis were performed by live examiner (KN) and co-authors (KR, PB, and AK). Any discrepancies in scoring were resolved by discussion with an inter-rater reliability of 95%.

### Structural brain scans and voxel-based morphometry

Within 1 week of initial behavioral testing, LV2 underwent high resolution, whole-brain T1-weighted structural MRIs (MP-RAGE) using a 3T Siemens Magnetom Skyra MRI scanner located at The University of Arizona [1 mm isotropic voxels, field-of-view = 256 mm, matrix = 256 × 256, 176 axial slices, repetition time (TR) = 2000 ms, time to echo (TE) = 2.33 ms, acquisition time (TA) = 293 s, scan time = 386 s]. Comparable scans were obtained for the 17 control participants to use in the implementation of voxel-based morphometry (VBM) to identify regions of reduced gray matter volume in LV2. VBM was conducted with gray matter images modulated by Jacobian determinants using Statistical Parametric Mapping-12 software (Wellcome Department of Cognitive Neurology, London) (see Supplementary Methods for details). To increase accuracy of inter-participant alignment, non-linear deformation parameters were calculated with the high dimensional Diffeomorphic Anatomical Registration Through Exponentiated Lie Algebra (DARTEL).

**FIGURE 1:**
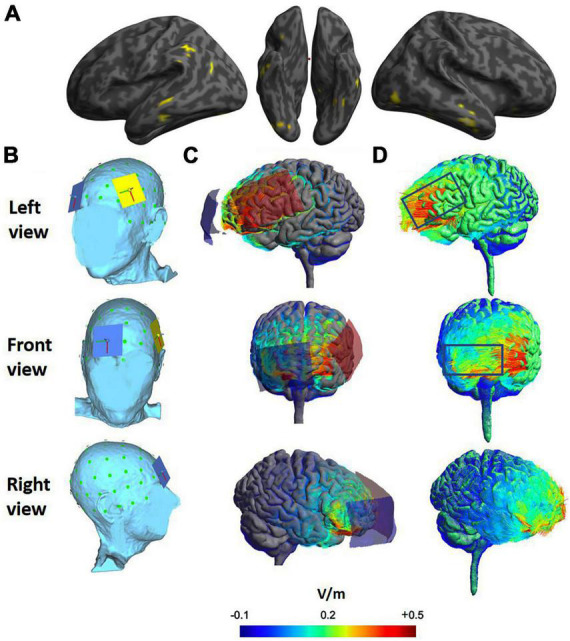
**(A)** Voxel-based morphometry showing regions of significant gray matter volume loss in yellow (thresholded voxel-wise at *p* < 0.001; corrected for family wise error at cluster level *p* < 0.05). **(B)** Position of anode (yellow over F5) and reference electrode (blue over Fp2). **(C)** Modeling of electrical fields induced by tDCS using SimNIBS. Current flow from anode (red) to cathode (blue) with 5 cm × 7 cm electrode/sponge shape depicted. **(D)** Magnitude of the induced electrical field. The directional fields normal to the cortical surface. The positive values (red lines) indicate inward field entering the cortical surface and negative values (blue lines) indicate outward electrical field leaving the surface.

Figure 1A shows significantly reduced gray matter volume in LV2 compared to healthy controls (voxel-wise threshold, *p* < 0.001, FWE cluster level, *p* < 0.05) in left posterior perisylvian language regions, including supramarginal gyrus and inferior parietal lobule (BA39/40), posterior superior temporal gyrus (BA22), middle and inferior temporal regions (BA 21/20), and the left fusiform gyrus (BA37). These cortical atrophy patterns are consistent with those commonly seen in lvPPA (Henry and Gorno-Tempini, 2010; Rohrer et al., 2013). Regions of significantly reduced gray matter volume were also detected in right middle temporal gyrus (BA21) and inferior temporal gyrus (BA 20), and right inferior occipital temporal cortex (BA18/19) (see Supplementary Table 1 for peak coordinates).

### Treatment

Based on her overall language profile, LV2 appeared to be a good candidate for behavioral treatment intended to strengthen phonological skills with the goal of providing stronger support for spoken and written language. Treatment was initiated in the week following pre-treatment assessment and implemented over two sequential 2-week phases (with five 60-min sessions/week = 10 h of treatment for each phase) separated by a 2-month interval with no treatment. As part of a larger study regarding the potential benefit of pairing tDCS with phonological treatment, LV2 was randomly assigned to receive active tDCS during the first treatment phase and sham tDCS during the second treatment phase. After another 2-month rest period, follow-up testing was administered.

Phonological treatment focused on sound-blending skills in a manner similar to the blending phase of treatment in previously reported cases of stroke-related phonological impairment (Beeson et al., 2010, 2018; DeMarco et al., 2018). Rather than training a specific set of targeted items to criterion, treatment focused on phonological manipulation skills across changing sets of stimuli. The goal was to improve performance on the following tasks: (1) sound blending (e.g., “Put these sounds together:/k/-/ae/-/t/”) expecting the response “cat”; (2) sound deletion (e.g., “Say ‘cat.’ Now take away/k/”) expecting “at”; (3) sound replacement for initial, medial, and final sounds (e.g., “Say ‘cat.’ Now change/k/to/f/) expecting “fat.” Training started with 1-syllable real words and progressed to 2-syllable real words, 2-syllable non-words, 3-syllable real words, and finally 3-syllable non-words. Within the treatment session, the clinician (KN) provided corrective feedback and increasing support as needed to achieve correct responses, including prompts to write the word/non-word to cue self-correction of spoken errors. The clinician offered guidance toward correct responses with the final (strongest) cue consisting of a model for spoken repetition, if needed. Treatment sessions typically involved training three to five novel stimulus items. Mastery at a given level was defined as 80% correct out of 5 untrained items on the blending task at the beginning of a treatment session. Thus, performance on the probe task prompted progression through the stimulus hierarchy (e.g., moving from 1-syllable words to 1-syllable non-words). Stimulus items for training and probes were unique and matched on relevant psycholinguistic properties (Supplementary Table 2).

All sessions were audio-recorded with on-line scoring reviewed by clinician and independently scored off-line by another author (FJ) who was also blinded to whether stimulation was active or sham. Inter-rater reliability (by item) was 90%, and any differences were resolved by consensus.

To document change over time, responses were tracked for probes on the three tasks: blending sounds for words, blending sounds for non-words, and reading non-words at each of the following time points: immediately before Phase 1 treatment (Pre1), after Phase 1 treatment (Post1), after a 2-month break (Pre2), then after Phase 2 treatment (Post2), and finally 2 months after treatment ended (Follow-Up). Comprehensive assessment measures were also repeated at those time points. Changes in the proportions correct over time were tested for significance using Chi-squared statistic with Yates correction (alpha level of 0.05).

### tDCS methods

Administration of tDCS was conducted with NeurConn1 Channel DC-Stimulator Plus (neuroCare Group, München, Germany) according to established guidelines (Brunoni et al., 2011; DaSilva et al., 2011; Bikson et al., 2016). Placement of electrodes on the scalp was determined according to the International 10/20 Electroencephalogram System (Homan, 1988; Okamoto et al., 2004; DaSilva et al., 2011). The anode was positioned over the left IFG (electrode F5) and cathode over the right supraorbital location (electrode FP2) for both active and sham phases of the intervention (Electrode positioning details in Supplementary Methods).

Decisions regarding electrode location, stimulation site, and polarity of the active electrode were informed by previous tDCS studies with individuals with PPA (e.g., Tsapkini et al., 2014, 2018; Cotelli et al., 2020; Nissim et al., 2020). LV2’s MRI scan and VBM results guided the identification of structurally healthy tissue in left frontal regions associated with phonological processing (Gold et al., 2005; Brunoni et al., 2011; Goranskaya et al., 2016). Prior to administration, we performed computational modeling of tDCS current flow to assess the likely area of effect using SimNIBS v3.0 with the finite element method (FEM, Saturnino et al., 2018, 2019; see Figures 1B–D). The head model was created from LV2’s high-resolution MRI scan (Supplementary Methods).

During treatment sessions with active tDCS (Phase 1), stimulation was delivered for 20 min at 1.5 mA using 5 cm × 7 cm saline soaked sponge electrodes (current density: 0.43 μA/mm^2^) with 15 s ramp-up and ramp-down periods. These parameters were chosen following previous tDCS studies showing positive effects on language performance (PPA: Gervits et al., 2016; Hung et al., 2017; Ficek et al., 2018; stroke: Baker et al., 2010; de Aguiar et al., 2015; healthy: Turkeltaub et al., 2012), with no significant adverse effects reported. Sham stimulation parameters were designed based on previous reports that the perceived sensations on the skin (e.g., tingling) fade out in the first 30 s of tDCS (Gandiga et al., 2006). During sham tDCS (Phase 2), current was ramped up for 15 s to 1.5 mA providing an initial sensation of tingling associated with active tDCS, and then ramped down to 0 mA over the last 15 s. The same 30-s procedure was repeated at the end of the 20-min period. This approach has been shown to successfully blind participants (de Aguiar et al., 2015; Darkow et al., 2017; Tsapkini et al., 2018). In addition to participant blinding, those involved in data collection and analysis did not know whether LV2 received active or sham tDCS. Adequacy of study blinding procedures was monitored after each session using questionnaires adapted from Brunoni et al. (2011). The safety of tDCS and any signs of participant discomfort were monitored continuously during treatment sessions. For both active and sham tDCS, LV2 rated discomfort on the following scale 1 = absent; 2 = mild; 3 = moderate; 4 = severe.

## Results

LV2 attended all treatment sessions (10 h total for each phase). She was unable to distinguish between active or sham tDCS, indicating “do not know” throughout the study when asked if she thought she received the “active” or “fake” stimulation. Similarly, the clinician also endorsed “don’t know” for both active and sham tDCS sessions. LV2 reported little to no discomfort during both active and sham tDCS, with average ratings of 1.1 (SD = 0.02).

### Treatment outcomes

**FIGURE 2:**
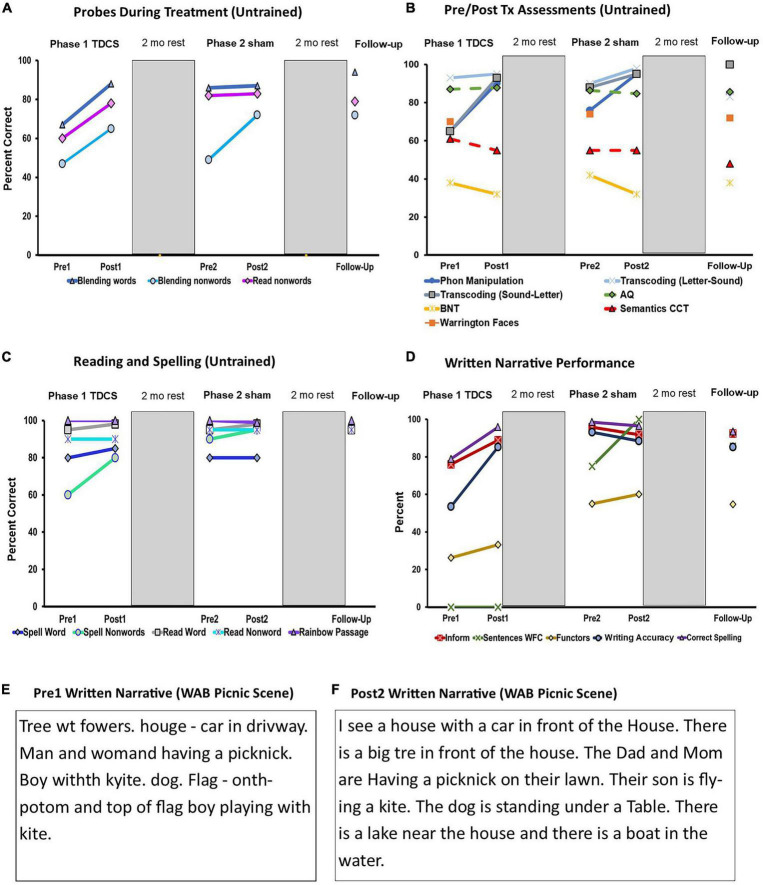
**(A)** Multiple baseline probes: Performance before and after treatment Phase 1 (active tDCS), Phase 2 (sham tDCS), and at follow-Up; **(B)** Pre/Post Tx assessment measures: Performance before and after treatment Phase 1 (active tDCS), Phase 2 (sham tDCS), and at follow-up for, AQ, aphasia quotient; BNT, boston naming test; CCT, Camel and Cactus Test; **(C)** Measures of reading and spelling: Performance before and after treatment Phase 1 (active tDCS), Phase 2 (sham tDCS), and at follow-up; **(D)** WAB written picture description: Performance before and after treatment Phase 1 (active tDCS), Phase 2 (sham tDCS), and at follow-up; Key: Informativeness (Inform) = [(#CIUs/#intelligible words)*100], Proportion grammatically well-formed sentences (Sentences WFC) = [(total well-formed sentences/total sentences)*100], Proportion correct functors (Functors) = [(total correct functors/total correct informational units)*100], Writing Accuracy = [#CIUs–(#spelling errors + #morphological errors)/(#words + #paragraphias)*100)], and Proportion CIUs spelled correctly (Correct Spelling) = [(CIUs-spelling errors/CIUs)*100]; **(E)** Transcript of LV2’s written picture description (WAB Picnic Scene) at pre-treatment Phase 1; **(F)** Transcript of LV2’s written picture description (WAB Picnic Scene) at post-treatment Phase 2.

During Phase 1 (active tDCS), LV2 progressed through the training protocol attaining 80% accuracy on 1-syllable stimuli after four sessions, 2-syllable stimuli after four sessions, and worked on 3-syllable stimuli during the last two sessions. Accuracy on the novel probe stimuli was documented for each syllable length, and average performance across syllable lengths is reported in Table 2 with significance determined using chi-squared statistics with Yates correction (alpha set at 0.05). As evident in Figure 2A, there was parallel improvement on the trained tasks after active tDCS: spoken blending of sounds significantly improved by 21% for words and 18% for non-words (details in Supplementary Table 3). Reading non-words aloud improved significantly by 18%. After the 2-month break (between Phase 1 and Phase 2), LV2 maintained performance for blending real words (86%) and reading non-words (82%), respectively. Non-word blending returned to initial level (49% correct) immediately before Phase 2 treatment. During treatment Phase 2 (sham tDCS), LV2 again progressed through the training hierarchy from 1-, 2-, and 3-syllable words across the three tasks in a manner comparable to Phase 1. After Phase 2, LV2 did not demonstrate additional gains in blending words or reading non-words aloud (maintained at 87% and 83%, respectively), but she made significant gains on blending non-words (72%) (Figure 2A). At the 2-month follow-up, she maintained gains on all three tasks; overall gains from initial testing (Pre-Treatment 1) to follow-up were significant as detailed in Table 2.

**TABLE 2:** Percent correct before and after treatment phases with active tDCS and sham tDCS, and at 2-month follow-up testing, with differences evaluated.

	Active tDCS (phase 1)				Sham tDCS (phase 2)				Post2–pre1		Follow-up		
	Pre1	Post1	Diff.	χ^2^	Pre2	Post2	Diff.	χ^2^	Diff.	χ^2^	FU	Diff.	χ^2^
**Probes during Tx**													
Blending words													
1-, 2-, and 3-syllable	67	88	**+21**	**11.5**	86	87	+1	0	**+20**	**10.2**	94	**+27**	**21.5**
Blending non-words													
1-, 2-, and 3-syllable	47	65	**+18**	**5.1**	49	72	**+23**	**10.1**	**+25**	**12.0**	72	**+25**	**12.0**
Read non-words													
1-, 2-, and 3-syllable	60	78	**+18**	**6.8**	82	83	+1	0	**+23**	**11.9**	79	**+19**	**7.6**
**Pre/post assessment**													
Phon manipulation**^1^**	65	90	**+35**	**16.5**	76	95	**+19**	**13.1**	**+30**	**26.3**	85	**+20**	**9.6**
Transcoding (L-Snd)**^1^**	93	95	+2	0.1	90	98	**+8**	**4.3**	+5	ns	83	**–10**	**3.8**
Transcoding (Snd-L)**^1^**	65	93	**+28**	**22.0**	88	95	+7	2.3	**+30**	**26.3**	100	**+35**	**40.0**
Aphasia quotient	87	88	+1	0	86	85	–1	0	–2	0.0	86	–1	0
Boston naming test	38	32	–6	0.6	42	32	–10	1.7	–6	0.6	38	0	0
Semantics (CCT)**^2^**	61	55	–6	0.5	55	55	0	0.0	–6	0.5	48	–13	2.9
Warrington faces	70				74						72	+2	0.0
**Spelling/reading**													
Spell word**^3^**	80	85	+5	0.6	80	80	0	0.0	0	0.0	95	**+15**	**9.0**
Spell non-words**^3^**	60	80	**+20**	**8.6**	90	95	+5	1.2	**+35**	**33.2**	95	**+35**	**33.2**
Read words**^3^**	95	98	+3	ns	95	98	+3	ns	+3	ns	95	0	0.1
Read non-words**^3^**	90	90	0	0.1	95	95	0	0.1	+5	1.2	95	+5	1.2
Rainbow passage	100	100	0	ns	100	99	–1	ns	0	ns	100	0	ns
**Written narratives**													
Informativeness	76	89	**+13**	**5.0**	96	92	–4	0.8	**+16**	**8.4**	93	**+17**	**9.8**
Correct spelling	79	96	**+17**	**11.7**	99	96	–3	ns	**+17**	**11.7**	94	**+15**	**8.4**
Writing accuracy	54	85	**+31**	**21.2**	93	89	–4	0.6	**+35**	**28.4**	85	**+31**	**21.2**
Sentences WFC**^4^**	0	0	0	ns	75	100	**+25**	**26.3**	**+75**	**116.9**	86	**+86**	**147.4**
Functors/CIUs	26	33	+7	3.9	55	60	+5	0.3	**+24**	**22.2**	55	**+29**	**16.3**

^1^Arizona phonological battery; L-Snd = Letter-to-Sound, Snd-L = Sound-to-Letter.

^2^Camels and Cactus Test.

^3^Arizona battery for reading and spelling.

^4^WFC, well-formed and complete. Chi-squared values with Yate’s correction; BOLD = significant improvement, *p* < 0.05; ns, not significant with expected cell frequencies 5 or less.

After completion of each treatment phase, the initial test battery was re-administered. Summary performance is shown in Table 2 and depicted for ease of comparison in Figure 2B (see Supplementary Table 3 for subtest data). After Phase 1 (active tDCS), LV2 showed significant improvement on phonological manipulation tasks from the Arizona Phonological Battery (APB, Beeson et al., 2010) (+35%) and sound-letter transcoding (+28%). When she returned from the 2-month break, she did not fully maintain those gains (Figure 2B) but was still above pre-treatment levels. After the second treatment phase (sham tDCS), she made significant gains on phonological manipulation (+19%) and letter-sound transcoding (+8%). At that time, performance was approaching ceiling for phonological manipulation (95%), letter-sound transcoding (98%), and sound-letter transcoding (95%). At the 2-month follow-up after treatment Phase 2, she showed some decline in accuracy, but the overall gain from before treatment Phase 1 to follow-up was +20% for phonological manipulation and +35% sound-letter transcoding. The latter improvement reflected resolution of allographic difficulty (i.e., letter selection errors) that was evident during initial testing. Transcoding of letters to sounds averaged 95% before any treatment and was maintained during the two treatment phases but declined to 83% after the 2-month interval to follow-up. LV2 made significant improvement in spelling non-words (+20%) during Phase 1, and she maintained those gains throughout (Figure 2C). At the start of Phase 2 treatment (real word spelling was 80% correct, and performance was 90% or better on other reading/spelling tasks (Figure 2C). There were no significant changes in reading/spelling after Phase 2, but at 2-month follow-up real word spelling improved to 95% correct, and non-word spelling was at 95%. Thus, overall changes from the initial pre-treatment performance were significant for spelling real words (+15%) and non-words (+35), and LV’s reading accuracy remained relatively high over the course of treatment.

As shown in Table 2 and Figure 2B, there were no significant changes on other repeated test measures over time, including overall language performance (WAB AQ), confrontation naming (BNT), non-verbal semantics (Camels and Cactus test), and episodic memory for faces (Warrington Faces). The Ravens Coloured Progressive Matrices (Raven et al., 2003) was administered immediately before Treatment Phase 2 (Supplementary Table 4), performing at the 4th percentile, and again at follow-up testing, when LV2’s score was comparable at the 3rd percentile (Smits et al., 1997).

#### Sentence-level writing skills

The most dramatic changes were observed in LV2’s written narratives (Table 2 and Figure 2D). After Phase 1 (active tDCS), the informativeness of her picture description increased significantly by 13%. Spelling of correct information units also improved by 17%, and the index of written word accuracy improved by 31%. The latter score reflected a greater proportion of correct information units that were without spelling or morphological errors in relation to all words written. At the end of Phase 1, LV2 still did not produce any well-formed, complete sentences; however, when she returned from the 2-month break, 75% of her written sentences met the criteria as well-formed and complete. At the end of treatment Phase 2, this increased significantly to 100%. Transcripts of SVs written narratives in Figures 2E, F show the contrast between pre-treatment 1 and post-treatment 2. Compare, for example, *car in driveway* (Figure 2E) from before treatment to *I see a house with a car in front of the house* (Figure 2F) written after treatment Phase 2. Two months following the completion of Phase 2 treatment, LV2 demonstrated maintenance of narrative writing skills, with performance significantly above baseline for informativeness, sentences well-formed and complete, proportion of functors, correct spelling, and overall writing accuracy. These improvements were observed in the context of significant decline in the informativeness of her spoken narratives (see Supplementary Table 5 and Supplementary Figure 1 for additional details).

#### Patient perspective

Over the course of intervention, LV2 reported greater confidence in her writing. She also shared that she had begun writing more at home (notes, emails, grocery lists). At the conclusion of treatment, she completed a self-rating questionnaire indicating her language performance was “much better” compared to before the intervention (5 on a 5-point scale). Her husband completed the Communication Effectiveness Index (Lomas et al., 1989) before and after treatment to provide his perception of LV2’s everyday communication activities. His overall ratings increased from 75% before treatment to 100% after Phase 1 and 92% after Phase 2. In a post-treatment interview, LV2 stated about treatment, “Really, it has changed my life.” Taken together, it was clear that both LV2 and her husband perceived a positive, meaningful difference from the treatment.

## Discussion

We learned several things from this treatment study. Consistent with the expected logopenic profile, our participant had significant anomia and some mild impairment of repetition. The underlying phonological deficit was better revealed by LV2’s difficulty with phonological manipulation tasks, and the functional correlate of this impairment was her marked difficulty writing at the sentence level. She demonstrated a pattern of phonological text agraphia characterized by omission of grammatical functors with little semblance of sentence structure in her written picture description. This pattern was consistent with that documented in several individuals recovering from stroke-related aphasia who showed persistent phonological text agraphia following relatively well-recovered spoken language (Beeson et al., 2018, 2019; DeMarco et al., 2018). In fact, LV2 similarly had better preserved spoken than written narratives. Like those with stroke-related impairment, LV2 improved phonological skills in response to behavioral treatment and showed dramatic generalization in her improved grammatical construction of written sentences. Thus, we learned that phonological impairment in lvPPA may present as phonological text agraphia, and importantly, that it responds to behavioral treatment designed to strengthen underlying phonological skills.

Over the course of treatment Phase 1 with active tDCS, LV2 showed significant improvements on the probes of phonological skill with relatively good maintenance after the 2-month rest. During the second phase of treatment with sham tDCS she continued to improve on the non-word blending task but leveled off for blending words and reading non-words, with performance over 80% correct. There was also significant improvement in phonological skills following phonological treatment with active tDCS and good maintenance during the sham phase. LV2 benefitted from sequential phonological treatments with active tDCS and sham in that the cumulative boost to phonological skills was reflected in written spelling (words and non-words) and production of written narratives. Most importantly, these gains were maintained 2 months after discontinuation of intervention (4 months and 2 weeks post active tDCS).

Examination of treatment gains over the full time-course of the study gives the impression of a particularly robust outcome after the first phase (active tDCS) and further consolidation of learning over Phase 2 (sham tDCS) and even during rest intervals. However, this single case does not allow us to disentangle the order effects and the fact that as LV2 improved, some tasks approached ceiling, leaving little room for improvement in some areas. Importantly, we demonstrated strong, generalized treatment outcomes and documented that LV2 tolerated tDCS administration and was unable to distinguish it from sham tDCS. She reported minimal discomfort associated with either active or sham tDCS. Taken together, these results support further examination of this treatment protocol in a double-blinded, randomized control study, which is in progress.

Given that evaluation of treatment efficacy for PPA is still in the early stages, it is worthwhile to consider the impact of the treatment reported here. The protocol implemented in this study was designed to improve the function of underlying phonological skills so that success would inherently generalize to untrained stimuli. Training was not item-specific, in fact, we went to great lengths to generate novel stimuli for every training session and every set of probe stimuli. Whereas most treatment studies examine outcomes for trained items and measure for generalization to untrained items, our protocol targeted performance on constantly changing stimuli, only measuring generalization effects. In addition, we documented generalization to language tasks that rely on phonological skill but were different from the treatment tasks, specifically, written spelling and sentence-level writing. We note that although the impact of phonological treatment was dramatic regarding written output, LV2 did not demonstrate changes in spoken modality. Her aphasia quotient and naming performance on the BNT remained about the same. In our view, continued treatment for LV2 might include strategy-based lexical retrieval cascade treatment to leverage improved phonological/orthographic skills (Henry et al., 2013).

Although we did not include extensive assessment of non-verbal cognitive processes, it was interesting to note that LV2 remained relatively stable. Given that treatment was implemented over the course of 5 months, the fact that she maintained performance rather than declining may reflect a protective effect of the treatment, but this is unclear. We will be able to address this in more detail in our larger study. Most importantly, we demonstrated that targeted intervention can improve functional language skills in progressive aphasia.

## Data availability statement

The original contributions presented in this study are included in the article/Supplementary material, further inquiries can be directed to the corresponding author.

## Ethics statement

The studies involving human participants were reviewed and approved by The University of Arizona. The patients/participants provided their written informed consent to participate in this study. Written informed consent was obtained from the individual(s) for the publication of any potentially identifiable images or data included in this article.

## Author contributions

KN and AK conceptualized the study, developed the treatment materials, and wrote the manuscript. PB and KR contributed to the conception and design of the study and helped write the manuscript. KN acquired behavioral data, administered behavioral treatment, and analyzed the data. AK acquired funding, contributed to the data analysis, and was responsible for all parts of the study. FJ acquired case data and assisted with tDCS administration. All authors approved the submitted version.
