# Supplementary material for: Positive changes to written language following phonological treatment in logopenic variant primary progressive aphasia: Case report
Source: Front Hum Neurosci. 2023 Jan 25;16:1006350. doi: 10.3389/fnhum.2022.1006350 (PMC9905434; doi:10.3389/fnhum.2022.1006350)
Supplement: Supplementary file 1 [file Data_Sheet_1.docx]

Supplementary Material

1. **Supplementary Methods**
   1. **VBM Methods**

Before processing, T1 images were evaluated for quality and were manually repositioned to set the anterior commissure as the origin to ensure consistent starting estimates for the unified segmentation routine. Segmented, spatially normalized, bias corrected, and smoothed gray matter maps were derived for all participants (Ashburner, 2007; Ashburner & Friston, 2005). To increase the accuracy of inter-participant alignment, a nonlinear deformation toolbox was used (Ashburner, 2007). For each individual, flow fields were calculated during template creation that contained the nonlinear deformation information for the native image transformation to the template, and the flow fields were applied to each participant’s image. The final template was registered to Montreal Neurological Institute (MNI) space using an affine transformation incorporated into the warping process to bring spatially normalized scans into common MNI space. During this final normalization step, the gray and white matter probability maps were scaled by their Jacobian determinants and smoothed using a 10 mm full width at half-maximum (FWHM) isotopic Gaussian kernel. LV2’s brain images were compared to the control group using an independent samples t-test, with age, sex, education, and total intracranial volume included as covariates.

**1.2. tDCS Methods**

**1.2.1. SimNIBS Modeling of tDCS current flow**

The goal of current flow modeling was to affirm polarization under each electrode, expecting mostly inward current under the anode and mostly outward current under the cathode. To perform head modeling the high resolution T1 scan was segmented into gray matter, white matter, cerebrospinal fluid (CSF), bone, scalp, eye region, muscle, and air cavities. The isotropic electrical conductivities (in S/m) were assigned as follows gray matter: 0.275; white matter: 0.126; CSF: 1.654; bone: 0.01; scalp: 0.465; eyeballs: 0.5; blood: 0.6, muscle: 0.160; air: 1e-15; sponge: 1.4; electrode rubber: 29.4; saline: 1.0.

- - 1. **Placement of tDCS electrodes**

LV2 was fitted with an EEG cap template with 10/20 standard electrode locations marked. The correct placement of the electrode cap on each session was guided by measuring head circumference and identifying locations of fiducial markers, the nasion, inion, two peri-auricular points and vertex Cz. Location Cz was identified at the intersection of the central line measured from nasion to inion and line from left to right preauricular points. The location of the target electrode was marked on the scalp using a red color china pencil. The same cap and fitting procedure were used for all treatment sessions. Because current generated by tDCS is broadly distributed the stimulated area was most likely larger than cortical tissue laying directly under F5. This is visualized in the SimNIBS current flow model (**Figure 1**).

**Supplementary Table S1**. Results of voxel-wise morphometry (VBM) analysis

| Brain Region |  | Brodmann’s  Area | MNI coordinates | Extend voxels | Max *t* | *p* |
| --- | --- | --- | --- | --- | --- | --- |
|  |  |  |  |  |  |  |
| Middle temporal gyrus | R | 21 | 58 -22 -14 | 31 | 4.00 | <.0001 |
| Middle temporal gyrus | L | 21 | -55 -27 -13 | 298 | 5.02 | <.0001 |
| Inferior temporal gyrus | R | 20 | 54 -13 -29 | 244 | 4.07 | <.0001 |
| Inferior temporal gyrus | L | 20 | -56 -4 -31 | 168 | 3.69 | 0.001 |
| Fusiform gyrus | R | 37 | 52 -63 -12 | 201 | 4.2 | <.0001 |
| Fusiform gyrus | L | 37 | -63 -50 -12 | 96 | 3.96 | <.0001 |
| Inferior occipital gyrus | R | 18/19 | 44 -84 -11 | 337 | 5.04 | 0.0001 |
|  |  |  |  |  |  |  |
| Lingual gyrus | R | 19 | 48 -70 -7 | 83 | 4.25 | <.0001 |
| Hippocampus/Parahippocampal | L | 28 | -26 -10 -21 | 9 | 3.81 | 0.001 |
| Angular gyrus | L | 39 | -54 -48 18 | 264 | 4.52 | <.0001 |
| Supra marginal gyrus | L | 40 | -57 -38 23 | 84 | 4.71 | <.0001 |
| Inferior parietal lobule | L | 40 | -48 -48 42 | 147 | 4.52 | 0.001 |

**Supplementary Table S2.** Psycholinguistics properties of pre- and post-treatment stimuli probes (with examples) for blending words, blending nonwords, and reading nonwords administered before and after each treatment phase

|  | **Blend Words** | |  | **Blend Nonwords** | |  | **Read Nonwords** | |
| --- | --- | --- | --- | --- | --- | --- | --- | --- |
| **Pre-Tx** | (bat) |  |  | (lut) |  |  | (tam) |  |
| **1-syllable** | *M* | *SE* |  | *M* | *SE* |  | *M* | *SE* |
| **Length_Syllable** | 1.00 | 0.00 |  | 1.00 | 0.00 |  | 1.00 | 0.00 |
| **Length_Phoneme** | 3.40 | 0.24 |  | 3.40 | 0.24 |  | 3.40 | 0.14 |
| **Length_# of Letters** | 3.40 | 0.24 |  | 3.40 | 0.24 |  | 3.40 | 0.14 |
| Frequency | 9.36 | 0.53 |  |  |  |  |  |  |
| Familiarity | 5.54 | 0.35 |  |  |  |  |  |  |
| Age of Acquisition | 4.55 | 0.35 |  |  |  |  |  |  |
| Imageability | 6.38 | 0.28 |  |  |  |  |  |  |
| **2-syllable** | (cabin) |  |  | (maset) |  |  | (nikot) |  |
| **Length_Syllable** | 2.00 | 0.00 |  | 2.00 | 0.00 |  | 2.00 | 0.00 |
| **Length_Phoneme** | 5.00 | 0.45 |  | 5.00 | 0.32 |  | 5.00 | 0.18 |
| **Length_# of Letters** | 5.20 | 0.37 |  | 5.20 | 0.49 |  | 5.00 | 0.18 |
| Frequency | 8.98 | 0.63 |  |  |  |  |  |  |
| Familiarity | 5.47 | 0.39 |  |  |  |  |  |  |
| Age of Acquisition | 4.97 | 0.59 |  |  |  |  |  |  |
| Imageability | 6.57 | 0.09 |  |  |  |  |  |  |
| **3-syllable** | (potato) |  |  | (denota) |  |  | (donlap) |  |
| **Length_Syllable** | 3.00 | 0.00 |  | 3.00 | 0.60 |  | 3.00 | 0.00 |
| **Length_Phoneme** | 6.00 | 0.00 |  | 6.00 | 1.20 |  | 7.40 | 0.29 |
| **Length_# of Letters** | 6.00 | 0.00 |  | 6.00 | 1.20 |  | 7.80 | 0.28 |
| Frequency | 7.57 | 0.30 |  |  |  |  |  |  |
| Familiarity | 6.50 | 0.06 |  |  |  |  |  |  |
| Age of Acquisition | 5.14 | 0.68 |  |  |  |  |  |  |
| Imageability | 6.68 | 0.04 |  |  |  |  |  |  |
| **Post-Tx** | (mug) |  |  | (vog) |  |  | (lug) |  |
| **1-syllable** | *M* | *SE* |  | *M* | *SE* |  | *M* | *SE* |
| **Length_Syllable** | 1.00 | 0.00 |  | 1.00 | 0.00 |  | 1.00 | 0.00 |
| **Length_Phoneme** | 3.40 | 0.24 |  | 2.80 | 0.24 |  | 3.40 | 0.24 |
| **Length_# of Letters** | 3.80 | 0.37 |  | 3.80 | 0.24 |  | 3.60 | 0.24 |
| Frequency | 10.09 | 0.40 |  |  |  |  |  |  |
| Familiarity | 6.30 | 0.22 |  |  |  |  |  |  |
| Age of Acquisition | 4.49 | 0.57 |  |  |  |  |  |  |
| Imageability | 6.54 | 0.17 |  |  |  |  |  |  |
| **2-syllable** | (panda) |  |  | (tunta) |  |  | (pasak) |  |
| **Length_Syllable** | 2.00 | 0.00 |  | 2.00 | 0.00 |  | 2.00 | 0.00 |
| **Length_Phoneme** | 5.00 | 0.32 |  | 4.40 | 0.24 |  | 5.00 | 0.32 |
| **Length_# of Letters** | 5.40 | 0.51 |  | 5.20 | 0.20 |  | 5.20 | 0.20 |
| Frequency | 7.29 | 0.53 |  |  |  |  |  |  |
| Familiarity | 5.49 | 0.50 |  |  |  |  |  |  |
| Age of Acquisition | 5.79 | 0.70 |  |  |  |  |  |  |
| Imageability | 6.50 | 0.24 |  |  |  |  |  |  |
| **3-syllable** | (corona) |  |  | (lepido) |  |  | (piboro) |  |
| **Length_Syllable** | 3.00 | 0.00 |  | 3.00 | 0.00 |  | 3.00 | 0.00 |
| **Length_Phoneme** | 6.00 | 0.00 |  | 5.40 | 0.24 |  | 7.40 | 0.51 |
| **Length_# of Letters** | 6.60 | 0.24 |  | 6.00 | 0.00 |  | 7.60 | 0.51 |
| Frequency | 7.36 | 0.62 |  |  |  |  |  |  |
| Familiarity | 4.76 | 0.23 |  |  |  |  |  |  |
| Age of Acquisition | 6.04 | 0.71 |  |  |  |  |  |  |
| Imageability | 6.53 | 0.05 |  |  |  |  |  |  |
| **T-tests Pre-Tx vs. Post-Tx** | ***t*** | ***p*** |  | ***t*** | ***p*** |  | ***t*** | ***p*** |
| **Length_Syllable** | 0.00 | 1.00 |  | -0.38 | 0.71 |  | 0.00 | 1.00 |
| **Length_Phoneme** | 0.00 | 1.00 |  | 0.60 | 0.56 |  | 0.00 | 1.00 |
| **Length_# of Letters** | -0.60 | 0.55 |  | -0.34 | 0.73 |  | 0.00 | 1.00 |
| Frequency | 0.99 | 0.33 |  |  |  |  |  |  |
| Familiarity | -0.02 | 0.98 |  |  |  |  |  |  |
| Age of Acquisition | -1.02 | 0.32 |  |  |  |  |  |  |
| Imageability | 0.17 | 0.87 |  |  |  |  |  |  |

Length_Syllable: syllable length, Length_Phoneme: number of phonemes, Length_# of Letters: number of letters; Frequency: Freq_HA, LOG10 version of frequency norms based on the Hyperspace Analogue to Language (HAL) corpus; Familarity: FAM_Glasgow, a word’s subjective familiarity on a scale 1(unfamiliar) to 7(familiar); Age of Acquisition: AoA_Kuperman12: The age at which people acquire the word, participants were asked to enter age in years at which they thought that they learned the word. Imageability: Glasgow scale: degree of effort involved in generating a mental image of the concept on a scale 1(unimageable) to 7(imageable). Psycholinguistic measures extracted from SCOPE South Carolina Psycholinguistic Metabase: <https://www.sc.edu/study/colleges_schools/artsandsciences/psychology/research_clinical_facilities/scope/search.php>

|  | **Active tDCS (Phase 1)** | | | |  | **Sham tDCS (Phase 2)** | | | |  | **Post2-Pre1** | |  | **Follow-Up** | | | |
| --- | --- | --- | --- | --- | --- | --- | --- | --- | --- | --- | --- | --- | --- | --- | --- | --- | --- |
|  | **Pre1** | **Post1** | **Diff** | ***χ2*** |  | **Pre2** | **Post2** | **Diff** | ***χ2*** |  | **Diff** | **χ2** |  | **FU** | **Diff** | ***χ2*** |  |
| **Probes during Tx** |  |  |  |  |  |  |  |  |  |  |  |  |  |  |  |  |  |
| ***Blending words*** |  |  |  |  |  |  |  |  |  |  |  |  |  |  |  |  |  |
| 1-syllable | 87 | 100 | **+13** | ***11.9*** |  | 93 | 93 | 0 | *.08* |  | +6 | *1.4* |  | 100 | **+13** | ***11.9*** |  |
| 2-syllable | 73 | 93 | **+20** | ***12.8*** |  | 93 | 93 | 0 | *.08* |  | **+20** | ***12.8*** |  | 93 | **+20** | ***12.8*** |  |
| 3-syllable | 40 | 70 | **+30** | ***17.0*** |  | 73 | 75 | +2 | *.03* |  | **+35** | ***23.7*** |  | 90 | **+50** | ***52.8*** |  |
| ***Blending nonwords*** |  |  |  |  |  |  |  |  |  |  |  |  |  |  |  |  |  |
| 1-syllable | 33 | 87 | **+54** | ***58.5*** |  | 73 | 93 | **+20** | ***12.8*** |  | **+60** | ***77.5*** |  | 67 | **+34** | ***21.8*** |  |
| 2-syllable | 47 | 67 | **+20** | ***7.4*** |  | 27 | 67 | **+40** | ***30.5*** |  | **+20** | ***7.4*** |  | 60 | +13 | *2.9* |  |
| 3-syllable | 60 | 40 | **-20** | ***7.2*** |  | 47 | 55 | +8 | 1.0 |  | -5 | *.33* |  | 90 | **+30** | ***22.4*** |  |
| ***Read nonwords*** |  |  |  |  |  |  |  |  |  |  |  |  |  |  |  |  |  |
| 1-syllable | 80 | 60 | **-20** | ***8.6*** |  | 80 | 73 | -7 | *1* |  | -7 | *1* |  | 87 | +7 | *1.31* |  |
| 2-syllable | 40 | 93 | **+53** | ***60.7*** |  | 93 | 100 | +7 | *ns* |  | **+60** | ***82.9*** |  | 80 | **+40** | ***31.7*** |  |
| 3-syllable | 60 | 80 | **+20** | ***8.6*** |  | 73 | 75 | +2 | *.03* |  | **+15** | ***4.5*** |  | 70 | +10 | *1.8* |  |
|  |  |  |  |  |  |  |  |  |  |  |  |  |  |  |  |  |  |
| **PostTx Assessment** |  |  |  |  |  |  |  |  |  |  |  |  |  |  |  |  |  |
| ***Phon Manipulation^1^*** |  |  |  |  |  |  |  |  |  |  |  |  |  |  |  |  |  |
| Sound Blending | 65 | 100 | **+35** | ***40.0*** |  | 85 | 100 | **+15** | ***14.1*** |  | **+35** | ***40.0*** |  | 95 | **+30** | ***26.3*** |  |
| Sound Replacement | 40 | 73 | **+33** | ***20.8*** |  | 54 | 87 | **+33** | ***24.6*** |  | **+47** | ***45.7*** |  | 63 | **+23** | ***9.7*** |  |
| Sound Segmentation | 90 | 98 | **+8** | ***5.7*** |  | 89 | 98 | **+9** | ***5.3*** |  | **+8** | ***5.7*** |  | 96 | +6 | *1.9* |  |
| ***Transcoding^1^*** |  |  |  |  |  |  |  |  |  |  |  |  |  |  |  |  |  |
| Letter – Sound | 95 | 100 | +5 | *ns* |  | 100 | 100 | 0 | *ns* |  | +5 | *NS* |  | 95 | 0 | *0.1* |  |
| Sound – Letter | 65 | 95 | **+30** | ***26.3*** |  | 95 | 95 | 0 | *0.11* |  | **+30** | ***26.3*** |  | 100 | **+35** | ***40.0*** |  |
| CVC NW Read | 90 | 90 | 0 | *0.1* |  | 80 | 95 | **+15** | ***9.0*** |  | +5 | *1.2* |  | 70 | **-20** | ***-11.3*** |  |
| CVC NW Write | 65 | 90 | **+25** | ***16.5*** |  | 80 | 95 | **+15** | ***9.0*** |  | **+30** | ***26.3*** |  | 100 | **+35** | ***40.0*** |  |
| ***Reading & Spelling^2^*** |  |  |  |  |  |  |  |  |  |  |  |  |  |  |  |  |  |
| Read Words | 95 | 98 | +3 | *ns* |  | 95 | 98 | +3 | *ns* |  | +3 | *ns* |  | 95 | 0 | *0.1* |  |
| Read Nonwords | 90 | 90 | 0 | *0.1* |  | 95 | 95 | 0 | *0.11* |  | +5 | *1.2* |  | 95 | +5 | *0.1* |  |
| Spell Word | 80 | 85 | +5 | *0.6* |  | 80 | 80 | 0 | *0.03* |  | 0 | *0.0* |  | 95 | **+15** | ***9.0*** |  |
| Spell Nonwords | 60 | 80 | **+20** | ***8.6*** |  | 90 | 95 | +5 | *1.15* |  | **+35** | ***33.2*** |  | 95 | **+35** | ***33.2*** |  |

**Supplementary Table S3.** Percent correct before and after treatment phases with active tDCS and sham tDCS, and at 2-month follow-up testing, with differences evaluated using Chi-squared values with Yate’s correction.

^1^Arizona Phonological Battery, ^2^Arizona Battery for Reading and Spelling
**BOLD** = significant improvement, p<.05; ns = not significant with expected cell frequencies 5 or less.

**Supplementary Table S4.** Performance before and after treatment phases with active tDCS and sham tDCS, and at 2-month follow-up testing, with differences evaluated using Chi-squared values with Yate’s correction.

|  | **Active tDCS (Phase 1)** | | | |  | **Sham tDCS (Phase 2)** | | | |  | **Post2-Pre1** | |  | **Follow-Up** | | |
| --- | --- | --- | --- | --- | --- | --- | --- | --- | --- | --- | --- | --- | --- | --- | --- | --- |
|  | **Pre1** | **Post1** | **Diff** | ***χ2*** |  | **Pre2** | **Post2** | **Diff** | ***χ2*** |  | **Diff** | **χ2** |  | **FU** | **Diff** | ***χ2*** |
| **Overall Language** |  |  |  |  |  |  |  |  |  |  |  |  |  |  |  |  |
| ***WAB-R Subtests(%)^1^*** |  |  |  |  |  |  |  |  |  |  |  |  |  |  |  |  |
| Content | 100 | 100 | 0 | *0* |  | 100 | 90 | **-10** | ***8.5*** |  | **-10** | ***8.5*** |  | 100 | 0 | *0* |
| Fluency | 90 | 90 | 0 | *.1* |  | 90 | 90 | 0 | *.1* |  | 0 | *.1* |  | 90 | 0 | *.1* |
| Comprehension | 94 | 95 | +1 | *0* |  | 94 | 97 | +3 | *ns* |  | +3 | *ns* |  | 95 | +1 | *0* |
| Repetition | 88 | 94 | +6 | *1.5* |  | 88 | 92 | +4 | *.5* |  | +4 | *.5* |  | 90 | +2 | *.1* |
| Naming | 70 | 62 | -8 | *1.1* |  | 58 | 60 | +2 | *.0* |  | -10 | *1.8* |  | 68 | -2 | *.0* |
| **Repetition (%)** |  |  |  |  |  |  |  |  |  |  |  |  |  |  |  |  |
| CNRT**^2^** | 88 | 88 | 0 | *.1* |  | 94 | 93 | -1 | *0* |  | +5 | *1* |  | 93 | +5 | *1* |
|  |  |  |  |  |  |  |  |  |  |  |  |  |  |  |  |  |
| **Allographic Performance (%)** |  |  |  |  |  |  |  |  |  |  |  |  |  |  |  |  |
| Case Conversion | *nt* | 90 |  |  |  | 87 | 83 | -4 | *.4* |  |  |  |  | 85 | -5 |  |
|  |  |  |  |  |  |  |  |  |  |  |  |  |  |  |  |  |
| **Nonverbal Intelligence (%)** |  |  |  |  |  |  |  |  |  |  |  |  |  |  |  |  |
| RCPM**^3^** | *nt* | *nt* |  |  |  | 46 | *nt* |  |  |  |  |  |  | 38 |  |  |
|  |  |  |  |  |  |  |  |  |  |  |  |  |  |  |  |  |
| **Digit Span** |  |  |  |  |  |  |  |  |  |  |  |  |  |  |  |  |
| Forward | 6 | 7 | +1 |  |  | 7 | 6 | -1 |  |  | 0 |  |  | 6 | 0 |  |
| Backward | 4 | 5 | +1 |  |  | 3 | 4 | +1 |  |  | 0 |  |  | 4 | 0 |  |

**BOLD** values = significant improvement, p<.05; ns = not significant with expected cell frequencies 5 or less; nt = not tested. WAB-R = Western Aphasia Battery-Revised; CNRT = Children’s Nonword Repetition Test; RCPM = Raven’s Coloured Progressive Matrices (Abbreviated Version)

|  |  | **Active tDCS Phase 1** | | |  | **Sham tDCS Phase 2** | | |  | **Follow-Up** | |  |
| --- | --- | --- | --- | --- | --- | --- | --- | --- | --- | --- | --- | --- |
|  |  | **Pre1** | **Post1** | **Diff** |  | **Pre2** | **Post2** | **Diff** |  | **FU** | **Diff** |  |
| # of Words | Written | 25 | 27 | -2 |  | 72 | 61 | -9 |  | 67 | +42 |  |
|  | Spoken | 128 | 159 | +31 |  | 341 | 217 | -124 |  | 431 | +303 |  |
| # of CIUs | Written | 19 | 24 | +5 |  | 69 | 56 | -13 |  | 62 | +43 |  |
|  | Spoken | 61 | 75 | +14 |  | 159 | 88 | -71 |  | 132 | +71 |  |
| **Spoken Narrative (%)** |  |  |  |  | ***χ2*** |  |  |  | ***χ2*** |  |  | ***χ2*** |
| Informativeness |  | 48 | 47 | -1 | *0* | 47 | 41 | -6 | *.5* | 31 | **-17** | ***5.4*** |
| Function words/#CIUs |  | 72 | 68 | -4 | *.2* | 68 | 68 | 0 | *.0* | 61 | -11 | *2.2* |

**Supplementary Table S5.** Performance on the WAB picture description (spoken and written) during Phase 1 (phonological treatment + active tDCS), Phase 2 (phonological treatment + sham), and at 2 months follow-up
**Informativeness** = [(#CIUs/#intelligible words)*100]; **Functors** = [(total correct functors/total correct informational units)*100]; Chi-squared values with Yate’s correction; **BOLD** = significant improvement, *p*<.05

**F. Post2 Written Narrative (WAB Picnic Scene)**

**Supplementary Figure S1.** Transcripts of spoken and written narratives (WAB Picnic Scene) at Pre-Treatment Phase 1 (Pre1) and Post-Treatment Phase 2 (Post2)

**B. Written Narrative (Post2)**

1. **Written Narrative (Pre1)**


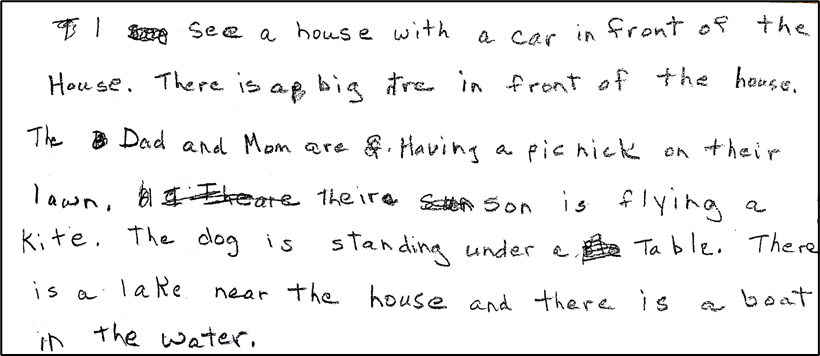

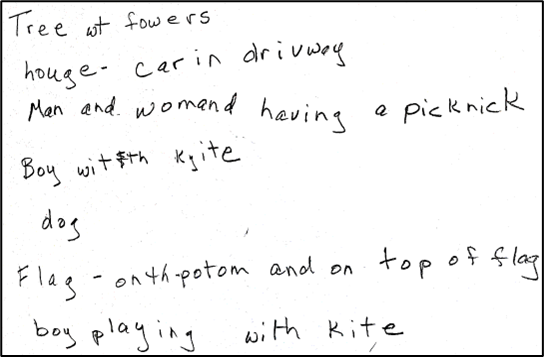


**C. Spoken Narrative Transcript (Pre1)**

**D.** **Spoken Narrative Transcript (Post2)**

This is a picture of a house. And the house has a car. And, in front of it, and there is a, um, tree in front of the house. And then there is what looks like a father and a mother. And they are sitting on the ground. And it looks like they might be having a picnic or some kind of a lunch. And, um, and then they have a low, a high, um… oh, on their house, they have this long- [Okay. Alright. We're gonna leave that for right this moment.] And, um, the Mom and Dad have a son. And he likes to play with his, um, with his kite. And, um, he also likes to watch the water. Um, and there is, um, a kind of [um, I'm gonna come back to that.] Um, okay, so then there is also a puppy or dog on the, um, on the dog with the boy. And, um, and then there's obviously a lake or little river that is beyond the house. And there is something going on here, but I can't really- I don't really understand what they're trying to tell me here. This looks like the boy. But this is [ I'm not doing well].[ Um, and I think that's the majority of what I have been seeing.]

It looks like, um, the family is having a picnic together. And, um, maybe a husband and a wife. And here is one of the children who’s playing with the kite. And then it looks like somebody’s on a boat over here. Um, and here’s the house ‘cause there’s the car. And here’s, here’s where they have a, um, [Can I come up with the word?] Here they have a, um, … [Oh, we were just talking about what what can you remember.] um, um… [Let’s see… ] um A flag, it's a flag, I think. No, um, that’s a flag. And, um, this is a tree with a lot of beautiful growth on it. And there’s a little doggie down here. [I don't, did I leave him out?]

**References:**

1. Ashburner, J., & Friston, K.J. (2005). Unified segmentation. Neuroimage. 26:839-851. doi: 10.1016/j.neuroimage.2005.02.018

2. Ashburner, J. (2007). A fast diffeomorphic image registration algorithm. Neuroimage. 38:95-113. doi: 10.1016/j.neuroimage.2007.07.007

**
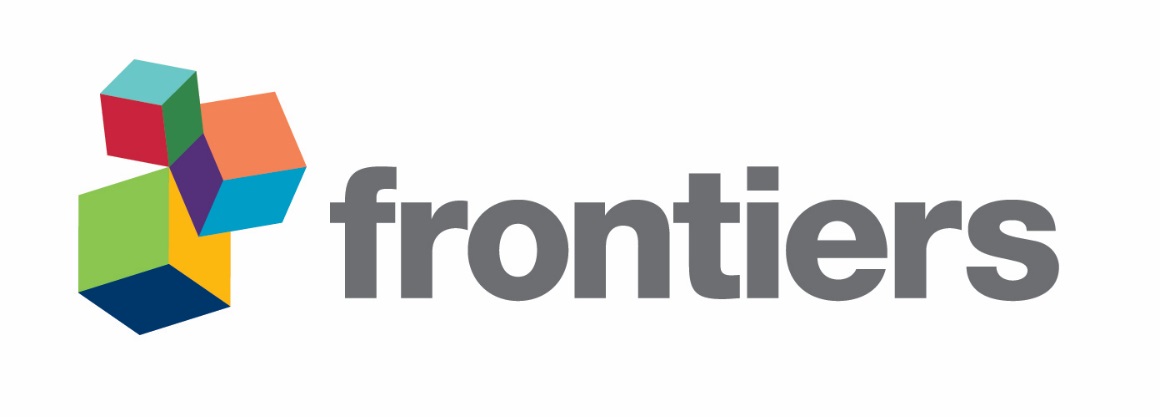
**
